# Pattern of medical waste management: existing scenario in Dhaka City, Bangladesh

**DOI:** 10.1186/1471-2458-8-36

**Published:** 2008-01-26

**Authors:** M Manzurul Hassan, Shafiul Azam Ahmed, K Anisur Rahman, Tarit Kanti Biswas

**Affiliations:** 1 Department of Geography and Environment, Jahangirnagar University, Savar, Dhaka, Bangladesh; 2 Water and Sanitation Programme, World Bank, Agargaon, Dhaka, Bangladesh; 3 Prism Bangladesh, House: 49, Road: 4/A, Dhanmondi, Dhaka, Bangladesh

## Abstract

**Background:**

Medical waste is infectious and hazardous. It poses serious threats to environmental health and requires specific treatment and management prior to its final disposal. The problem is growing with an ever-increasing number of hospitals, clinics, and diagnostic laboratories in Dhaka City, Bangladesh. However, research on this critical issue has been very limited, and there is a serious dearth of information for planning. This paper seeks to document the handling practice of waste (e.g. collection, storage, transportation and disposal) along with the types and amount of wastes generated by Health Care Establishments (HCE). A total of 60 out of the existing 68 HCE in the study areas provided us with relevant information.

**Methods:**

The methodology for this paper includes empirical field observation and field-level data collection through inventory, questionnaire survey and formal and informal interviews. A structured questionnaire was designed to collect information addressing the generation of different medical wastes according to amount and sources from different HCE. A number of in-depth interviews were arranged to enhance our understanding of previous and existing management practice of medical wastes. A number of specific questions were asked of nurses, hospital managers, doctors, and cleaners to elicit their knowledge. The collected data with the questionnaire survey were analysed, mainly with simple descriptive statistics; while the qualitative mode of analysis is mainly in narrative form.

**Results:**

The paper shows that the surveyed HCE generate a total of 5,562 kg/day of wastes, of which about 77.4 per cent are non-hazardous and about 22.6 per cent are hazardous. The average waste generation rate for the surveyed HCE is 1.9 kg/bed/day or 0.5 kg/patient/day. The study reveals that there is no proper, systematic management of medical waste except in a few private HCE that segregate their infectious wastes. Some cleaners were found to salvage used sharps, saline bags, blood bags and test tubes for resale or reuse.

**Conclusion:**

The paper reveals that lack of awareness, appropriate policy and laws, and willingness are responsible for the improper management of medical waste in Dhaka City. The paper also shows that a newly designed medical waste management system currently serves a limited number of HCE. New facilities should be established for the complete management of medical waste in Dhaka City.

## Background

Medical waste, due to its content of hazardous substances, poses serious threats to environmental health [1,2]. The hazardous substances include pathological and infectious material, sharps, and chemical wastes [3-6]. In hospitals, different kinds of therapeutic procedures (i.e. cobalt therapy, chemotherapy, dialysis, surgery, delivery, resection of gangrenous organs, autopsy, biopsy, para clinical test, injections etc.) are carried out and result in the production of infectious wastes, sharp objects, radioactive wastes and chemical materials [7]. Medical waste may carry germs of diseases such as hepatitis B and AIDS. In developing countries, medical waste has not received much attention and it is disposed of together with domestic waste [8,9]. Improper medical waste management is alarming in Bangladesh and it poses a serious threat to public health.

Medical waste contains highly toxic metals, toxic chemicals, pathogenic viruses and bacteria [10-12], which can lead to pathological dysfunction of the human body [13,14]. Medical waste presents a high risk to doctors, nurses, technicians, sweepers, hospital visitors and patients due to arbitrary management [15,16]. It is a common observation in Dhaka City that poor scavengers, women and children collect some of the medical wastes (e.g. syringe-needles, saline bags, blood bags etc.) for reselling despite the deadly health risks. It has long been known that the re-use of syringes can cause the spread of infections such as AIDS and hepatitis [17]. The collection of disposable medical items (particularly syringes), its re-sale and potential re-use without sterilization could cause a serious disease burden [18].

The safe disposal and subsequent destruction of medical waste is a key step in the reduction of illness or injury through contact with this potentially hazardous material, and in the prevention of environmental contamination [19]. The transmission of blood-borne viruses and respiratory, enteric and soft tissue infections through improper medical waste disposal is not well described [7]. The management of medical waste therefore, has been of major concern due to potentially high risks to human health and the environment [20,21].

The growing number of hospitals, clinics, and diagnostic laboratories in Dhaka City exerts a tremendous impact on public health and environment. All of the hospitals, clinics, and diagnostic laboratories are considered here as the HCE. Some 600 HCE in Dhaka city generate about 200 tons of waste a day [22]. Like ordinary household waste, medical wastes are generally dumped into DCC bins. It is reported that even body parts are dumped on the streets by the HCE. The liquid and solid wastes containing hazardous materials are simply dumped into the nearest drain or garbage heap respectively.

Proper management of medical waste is crucial to minimise health risks. The improvement of present waste management practices for HCE in Bangladesh will have a significant long-term impact on minimising the spread of infectious diseases. Medical waste requires specialized treatment and management from its source to final disposal. Simply disposing of it into dustbins, drains, and canals or finally dumping it to the outskirts of the City poses a serious public health hazard. Thus, there is a need to initiate a concentrated effort to improve the medical waste management to reduce the negative impact of waste on: (a) environment; (b) public health; and (c) safety at health care facilities.

There are different types of medical waste management systems in different countries [3,4,9,23-26]. Although, medical waste disposal options are not completely risk-free, the risks can be minimized with care [27]. Improper disposal of medical waste may include damage to humans by sharp instruments, diseases transmitted to humans by infectious agents, and contamination of the environment by toxic and hazardous chemicals [28-30]. Therefore, proper management of medical waste is a subject of major concerns for a healthy environment. In Bangladesh, medical waste management systems to reduce the environmental and public health risk are grossly inadequate [31,32].

Medical wastes account for a very small fraction, about one percent of the total solid wastes generated in Bangladesh [31]. However, when this tiny amount is not handled properly, it gets mixed with domestic solid waste, and the whole waste stream becomes potentially hazardous. Until recently, there was no system for proper medical waste management in Bangladesh to protect environmental health hazards. It was generally disposed of in the same way as ordinary domestic waste. But, very recently, government is trying to develop a system to handle medical waste properly. This paper seeks to document an inventory of different HCE in Dhaka City and to quantify the amount of wastes generated by each HCE. In addition, the paper presents the current waste handling practices in terms of storage, collection, transportation and disposal within and outside hospital premises.

## Methods

An extensive questionnaire survey provided breadth of coverage, while in-depth interviews with nurses and different respondents in HCE allowed a greater understanding of the waste management system within each surveyed hospital, clinic, and diagnostic centre. The collected data for this study were analysed to address the central issues of hospital waste management in relation to the generation of wastes from different sources.

### Study sites

Two municipal administrative wards (Wards 49 and 56) of Dhaka City were selected for this study. Dhanmondi (Ward 49), once a quiet residential area, was zoned for commercial establishments by the RAJUK (*Rajdhani Unnayan Katripakkha*, Capital Development Authority) in 1972. As a result, the number of hospitals, clinics and diagnostic centres in Dhanmondi has been increasing steadily. These HCE indiscriminately dump medical waste around their premises. Many poor children and adults salvage these items. Dhanmondi is a densely populated area and the number of HCE in Dhanmondi is the highest of all the administrative wards in Dhaka City.

**Figure 1:**
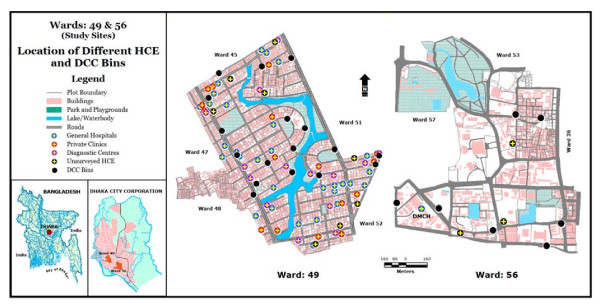
The study area and location of different HCE and DCC bins in the study sites. Almost all the HCE dispose their generated waste into the DCC bins located close to them.

Dhaka Medical College Hospital (DMCH), located in Ward 56, is the largest hospital in Bangladesh and creates a lot of waste. Therefore, Dhanmondi and the DMCH were selected to investigate the situation of medical waste generation and the existing management scenario (Figure 1). High population density in the study sites assures that a large number of people are exposed to toxic level of medical waste. In addition, school children are considered to be most vulnerable because the study sites are mainly located close to schools.

### Data

No statistically rigorous sampling procedure could be followed for this study. Those HCE who were willing to provide us information were selected for this study. It was not easy to collect relevant medical waste data from HCE since a number of HCE did not follow the existing rules and regulations to run them properly. This is why some HCE authorities were not interested to give permission in collecting data from their own institutions. A total of 60 out of existing 68 HCE in the study site gave us permission to collect waste data. Accordingly, we collected our data from these 60 HCE. Before entering a HCE, we arranged a number of formal meetings with the concerned authority of each HCE to explain the purpose of the study and seek their cooperation. After receiving consent, we started our fieldwork. A number of face-to-face formal and informal approaches were adopted in order to gather data.

In collecting our data, questionnaire surveys and in-depth interviews were adopted. Apart from this, the dialectic approach was used to confirm the credibility of stories and examine the 'cross-case themes' [33] that we gathered from in-depth interviews. The term 'dialectic' is a set of questions for trying to understand the empirical reality rather than a set of pre-designed answers [34]. This approach presents a way of thinking about intercultural communication that allows for a very rich understanding [35].

A questionnaire was designed following the objectives of this study. A total of 139 questionnaires (61 from DMCH, 59 from Ward 49, and 19 from BMCH) were surveyed. The questionnaire mainly addressed the issues of: (a) types of generated medical wastes; (b) sources of medical wastes; (c) amount of generated wastes; and (d) existing waste management practices. The occupation segmentation with gender heterogeneity was considered to select our respondents. Among the interviewees, 63 (45%) were female and 76 (55%) were male respondents. All of the female respondents were nurses, and the rest were doctors, medical technicians, and cleaners. The average age of the respondents was 39 years. Apart from the formal questionnaire survey, we had informal discussions with patients for their understanding about existing medical waste management.

The waste generation rates in the surveyed HCE were obtained by actual measurements and through assessment of the storage facilities emptying frequencies and the degree of filling of the refuse receptacles. Different wastes were used to store in different sizes of buckets in different wards, and surgery departments. The weight of these wastes was then measured in kilograms. The quantity of waste in different categories was collected twice in a day when the refuse receptacles emptied, but, in surgery departments, nurses provided us additional information regarding the quantity of generated wastes. We were very careful about our data and nurses were helpful in gathering the relevant information. The WHO guidelines were followed to categorize the generated wastes from different HCE in the study sites [7].

A number of in-depth interviews were arranged to get a greater understanding of the existing management practice of medical wastes. In-depth interviewing is a highly personal process where meanings are created through personal interaction [36-38]. A number of specific questions were asked of nurses, hospital managers, doctors, and cleaners for eliciting their understandings.

### Analysis

The data collected by questionnaire survey were analysed mainly with simple descriptive statistics; while the qualitative mode of analysis was mainly narrative. This method was adopted for the qualitative abstraction and vivid presentation of a new understanding of present medical waste management practices. The spatial data – point features of the HCE and DCC bins – were analysed with GIS for presentation of the existing spatial pattern of HCE and their final waste management in DCC bins. The distribution pattern of different surveyed HCE and DCC bins is helpful in describing the practice of medical waste dumping into the DCC bins as part of the existing management system.

## Results

### Inventory of HCE

The relevant information collected for this study were from general hospitals (**30**, 50.9%), private clinics (**15**, 25.4%), and diagnostic centres (**14**, 23.7%). Our field survey shows that the surveyed general hospitals, excluding the BMCH, offer medical facilities for about 600 resident patients and about 1,700 outpatients; the private clinics provide medical treatments for about 300 resident patients, and the diagnostic centres provide pathological facilities for about 1,900 patients (Table 1). The BMCH is the largest private hospital in Bangladesh and offers medical treatments for about 300 resident patients and 750 outpatients per day. Moreover, DMCH providing medical facilities for 1,700 resident patients and about 3,500 outpatients per day with pathology, radiology and imaging, microbiology, surgery, pharmacology and therapeutics, gynaecology, etc. (Table 1).

**Table 1:** Waste generation rates in surveyed hospitals

**HCE**	**Patients**		**Total patients**	**Waste generation rate**		
			
	**Beds**	**Outpatients**		**Kg/day**	**Kg/bed/day**	**Kg/patient/day**
DMCH	1700	3500	5200	3274	1.93	0.63
BMCH	300	750	1050	673	2.24	0.64
**Surveyed hospitals (Ward: 49)**						
- General Hospitals	591	1698	2289	943	1.60	0.41
- Private Clinics	293	-	293	380	1.30	-
- Diagnostic Centres	-	-	1867	292	-	0.16
**All surveyed HCE**	**2884**	**5948**	**10699**	**5562**	**1.93**	**0.52**

**Source: **Field survey, 2006

### Sources and Quantification

The amount of waste generated in hospitals depends upon various factors such as the number of beds, types of health services provided, economic, social and cultural status of the patients and the general condition of the area where the hospital is situated [3]. During our field survey, it was observed that the surveyed HCE generated pathological wastes, textiles stained with blood, cotton pads, used syringes, broken bottles and glasses, paper, cans and other metals, vegetable/rubbish, and sharps (syringe-needles, surgical blades and blood lancets).

Together the surveyed HCE generate 5,562 kg/day of wastes, of which 4,308 kg/day (77.4%) are general wastes and 1,254 kg/day (22.6%) are hazardous wastes (Table 2). Our field survey reveals that the average waste generation rate for all the surveyed HCE on the basis of beds available is 1.9 kg/bed/day (Table 1). The results compare with the medical waste generation rates reported to be 2.7 kg/bed/day in hospitals of Tehran (Iran) [39]; between 0.84 and 5.8 kg/bed/day in hospitals in Dar-es-Salaam (Tanzania) [23]; and between 4.5 and 9.1 kg/bed/day in US hospitals, of which about 10 per cent is thought to be infectious or disease-causing [6]. Kitchen wastes and other non-hazardous wastes were found to be the highest component in all the HCE in Dhaka and the quantity covers more than three-quarters of the generated wastes, followed by infectious wastes (14.1%), plastic wastes (3.8%), liquid wastes (3.4%), and sharp items (1.2%) (Table 2).

**Table 2:** Amount of wastes with types generated in all surveyed HCE

**Colour**	**Type of wastes**	Amount (in kg)*					
		
		**DMCH**	**BMCH**	GH**	PC**	DC**	**Total**
Black	General waste (Kitchen waste, medicine box)	2587 (79.01)	563 (83.65)	729 (77.31)	286 (75.26)	143 (48.97)	4308 (77.45)
Yellow	Infectious waste (Cotton bandage, amputated body parts, placenta, blood & urine bags)	489 (14.94)	59 (10.57)	132 (14.00)	46 (12.10)	57 (19.52)	783 (14.08)
Green	Plastic waste (Syringe without needle, saline bags, gloves)	79 (2.41)	18 (3.22)	32 (3.39)	21 (5.53)	63 (21.57)	213 (3.83)
Red	Sharp items (needle, blade, knife, Vial-ampoule)	36 (1.10)	6 (1.07)	12 (1.27)	9 (2.37)	6 (2.06)	69 (1.24)
Blue	Liquid waste	83 (2.53)	27 (4.83)	38 (4.03)	18 (4.74)	23 (7.88)	189 (3.40)
	**Total:**	**3274 **(100%)	**673 **(100%)	**943 **(100%)	**380 **(100%)	**292 **(100%)	**5562 **(100%)

**Source: **Field survey, 2006

* Figures in the parentheses indicate the per cent of each HCE category.

** **GH: **General Hospitals; **PC: **Private Clinics; and **DC: **Diagnostic Centres.

¤ For DC, Syringe (without needle) for injection and blood sampling was considered to be a hazardous element.

## Discussion

### Medical Waste Management: A Recent Past Scenario

**Figure 2:**
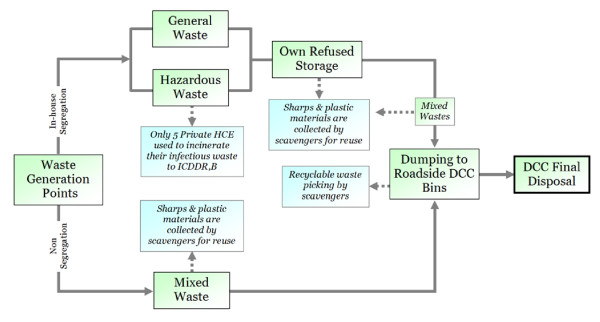
Previous waste management practice in Dhaka City, particularly before December 2005.

Until recently (December 2005), there has been an improper procedure of medical waste management in Dhaka City. No HCE segregated their generated wastes, except a very few. Medical wastes need to be segregated separately according to their characteristics at the point of generation [7]. In some HCE, all the infectious wastes were found to be separated from the non-infectious waste stream at the site of production, but during disposal in the DCC dustbins the wastes were then mixed together. In all of the HCE, pharmaceutical wastes and pressurized containers (e.g. inhalers, spray cans etc.) were disposed along with the general waste. The intermingling of infectious wastes with general waste in the HCE is a threat to environmental health. Figure 2 shows the previous medical waste management practices in Dhaka City.

Our field survey found that a very few private HCE used to collect their in-house waste systematically. They used to segregate their sharps and infectious wastes in separate bins and send off them to the International Centre for Diarrhoeal Disease and Research in Bangladesh (ICDDR, B) for incineration at the rate of Tk.70.00 ($1.00) per kg of waste. The two big hospitals in Dhaka City (DMCH and BMCH) generally were found to be disposing of their wastes into the DCC bins without segregating them. This poses serious health risks to the personnel handling the waste, to the scavengers at the dumpsite, and to the public at large. The consequences of this practice extend to the possibility of polluting both surface water and groundwater resources in the vicinity of dumpsite [4].

In the DMCH, generated wastes from gynaecology departments (e.g. sanitary napkins, liquid wastes, placenta, disposable gloves, etc.) and operation theatres were found to be collected in metal or plastic buckets for disposing into DCC bins without segregation. Amputated body parts (e.g. hands, legs, gall bladder, uterus, tumour, aborted foetus, and many others) generated from operation theatres were mainly disposed of in DCC bins by the sweepers and cleaners. Some cleaners were found to be engaged in mishandling the generated wastes within the HCE premises and at the DCC bins. They salvage used sharps (mainly the syringe-needles), saline bags, blood bags and test tubes for resale or reuse.

Internal and central storage facilities are important to store the collected waste for a certain period until safe disposal. Some small HCE do not have any temporary storage and they simply used to dispose the waste directly into the nearest DCC bin. Every morning, the DCC collects their bins from different roadsides and finally dumps at different waste disposal sites located outside the city boundary. Our field survey shows that all of the surveyed HCE dispose of their domestic waste at the same site as the municipal waste.

### New Approach of Medical Waste Management

There are a number of guidelines for the management of infectious waste materials from medical institutions [11,40-44]. Medical facilities in different HCEs in Bangladesh are characterised by inadequate and inappropriate refuse storage facilities, lack of refuse collection services, improper disposal methods and inadequate and inappropriate protective gear for refuse handlers. In Bangladesh, proper medical waste management is a new phenomenon and Government of Bangladesh is trying to develop a new and modern approach to deal with the medical waste properly.

**Figure 3:**
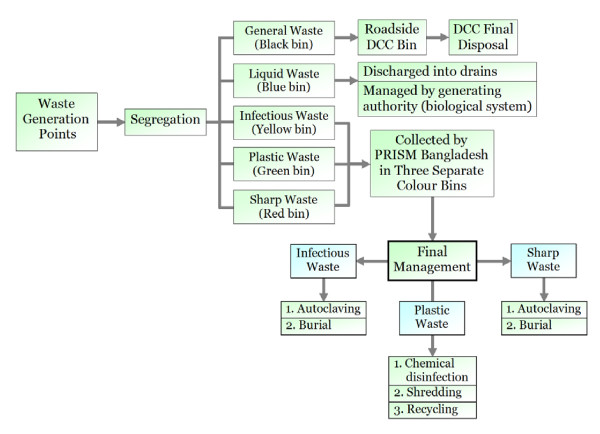
Existing waste management practice in some HCE Dhaka City (in-house waste segregation and final disposal) organised by PRISM Bangladesh. This new system has been practised since December 2005.

PRISM (Project in Agriculture, Rural Industry, Science and Medicine) Bangladesh, a reputed national NGO in Bangladesh, is now working for medical waste management in association with the DCC. With financial and technical support from Water and Sanitation Programme (WSP), PRISM Bangladesh carried out a survey on the medical waste management in Dhaka City. Subsequently, PRISM Bangladesh with the financial support from Canadian International Development Agency (CIDA) has recently developed a disposal facility for low cost medical waste treatment and management in Dhaka City. The DCC has allocated one acre (0.405 hectare) of land in Matuail, a dumpsite near the city limit for the final disposal of medical waste. It is inadequate to handle all the medical wastes of the city with the limited facilities of final disposal. PRISM Bangladesh is managing the generated medical waste in different forms (Figure 3).

In the new approach, PRISM Bangladesh is involved in training relevant personnel of different HCE for increasing awareness and proper in-house management of medical wastes. A public awareness campaign for proper management of medical waste would be effective in keeping up the city environment safe. Awareness is essential to solve this problem, particularly with regards to the reuse of syringes and needles and other sharps contaminated with human blood or body fluids. PRISM Bangladesh has recently provided training for more than 3000 personnel in 185 HCE.

PRISM Bangladesh has developed a system for collecting segregated hazardous wastes (except radioactive wastes) from each HCE through newly set up vehicles to carry this waste for final dumping at their newly developed Matuail Plant. It has introduced in-house storage of medical waste in colour-coded bins. Segregation of waste at source into suitable colour-coded bins is vital to a proper waste management. All of the HCE should now be using the Government of Bangladesh (GoB) approved colour-coded high-density polyethylene bags for easy identification and segregation of infectious and non-infectious medical wastes. This minimises the actual volume of potentially infectious medical waste and makes the disposal less costly and more effective.

The colour codes recently introduced by the GoB should be followed at all HCE for this purpose. Infectious waste should be packaged: (a) to protect waste handlers and the public from possible injury and disease that could result from exposure to waste; and (b) to avoid attracting rodents and vermin [9]. The integrity of packaging can be preserved during handling, storage, transportation and treatment. In all the surveyed HCE, sharp instruments are generally stored in separate refuse receptacles. The colour-coded waste segregation guide represents best practice and ensures, at minimum, compliance with current regulations. Some HCE in Dhaka City are paying a service charge for the collection from their premises and final management at the Matuail plant.

Reuse of syringes and needles is extremely harmful to human health. There was no system and/or practice of destroying needles from used syringes in the HCE in Dhaka in near past. To protect resale and reuse of syringes, both manual and electric needle destroyers have recently been introduced to different HCE to cut needles from syringes to protect against HIV and Hepatitis viruses. All of the generated plastic items (i.e. syringes without needles and intravenous bags) and glass materials (i.e. vial, ampoule, slide, broken glasses etc) are disinfected by immersion in different strength chlorine chambers. Then they are destroyed in a locally developed shredder machine to prevent reuse. The disinfecting and shredded plastic items are used for recycling. Moreover, a small effluent treatment plant has recently been constructed to treat the waste water generated in the plant. One heavy duty autoclaving machine has recently been installed for sterilizing cotton, gauge, bandage and such other contaminated materials. These sterilized materials are mixed up with the general civic waste for disposing in DCC dumping area. Organic infectious waste and sharp items are buried in the separate concrete burial pits at the newly constructed plant.

All hazardous materials except plastic and polymer materials can be incinerated. An incinerator donated recently by a Japanese organisation will be installed at the Matuail plant. The use of an incinerator treats a large amount of waste as well as reduces the volume of waste considerably. Our field survey found that the DMCH had installed two manually operated incinerators, but these incinerators have been out of order for the last five years. Patil & Pokhrel [9] are in favour of incineration; while Bakoglu *et al *[45] and Karademir [46] assess health risk of Polychlorinated Dibenzodioxin and Dibenzofuran (PCDD/F) emissions from a medical waste incinerator in Turkey. Maoa *et al *[47] also advocate against the incinerator because of its mass emission of particulate matter (PM2.5/PM10) and particle-bound polycyclic aromatic hydrocarbons (PAH), which is considered to be health risk. Although there have been a number of criticisms of incinerators regarding their impact on the environment, this incinerator has been shown to be an alternative technology in destroying infectious medical wastes. If an incinerator is operated properly (high and continuous temperature, filtration of particulate emission, disulfuration etc), it will not incur excessive risks and it can be affordable for medical waste management.

Our field survey shows that all the HCE discharge their liquid pharmaceutical and chemical waste into the general sewers or drains in Dhaka City because none of them have any proper liquid waste management facilities. Liquid waste is mainly generated from patients' service units, operation and surgical units, laboratories and other health-care units. All the HCE surrounding the Dhanmondi Lake in Ward 49 dispose of their liquid waste into this lake. Liquid waste generated from HCE is genotoxic and is normally polluted with Biochemical Oxygen Demand (BOD), Chemical Oxygen Demand (COD), Total Suspended Solids (TSS), faecal coliform, and total coliform content above tolerable limits [48]. The post mortem department of the DMCH generates hazardous wastes stained with blood, body fluids etc. Anatomical waste is disposed through burial; liquid wastes are disposed into general sewerage; and stained wastes are burned manually.

Our field survey shows that a poor coordination between two different ministerial agencies, Department of Environment (DoE) (under the Ministry of Environment and Forest, MoEF) and the DCC, has slowed down medical waste management. The DCC is mainly responsible for the operation of medical waste management, while the DoE is the Authority for ensuring the environmental law to be implemented. Thus, a good coordination between the two agencies is emphasized. In addition, the DCC should go for Public-Private Partnership (PPP), since the DCC has a limited capacity for proper handling of medical waste in Dhaka City.

There is no national policy on medical waste management in Bangladesh. For a proper and scientific management of medical waste, government should give priority to formulate a policy. Moreover, the existing laws are generally outdated and characterised by low penalties and sometimes no penalties at all for offenders. Thus, massive awareness towards this issue and formulating new tougher laws could be effective in protecting people and the environment from deadly medical waste. Recently a Law has been proposed to handle medical waste properly, but it needs to be adopted and enforced as soon as possible.

## Conclusion

Disposal of medical wastes is a growing environmental problem in Bangladesh. Until recently, the management of medical wastes has received little attention despite their potential environmental hazards and public health risks. The paper has attempted to quantify different medical wastes generated from different HCE in the study area. The surveyed HCE generated about 77.4% of non-infectious wastes and about 22.6% of infectious wastes. The average waste generation rate for the surveyed HCE is 1.9 kg/bed/day. Our field data shows that almost all the HCE do not segregate their generated wastes and they dispose of their domestic waste at the same site as normal civic waste.

The generation of medical waste in Dhaka has been increasing in quantity and variety, due to the wide acceptance of single-use disposalable items. In the recent past, medical waste was often mixed with household waste and disposed of in municipal solid waste landfills. In recent times, increased concerns over improper disposal of medical waste have led to a movement to regulate the waste more systematically. Efforts have to be made for minimization and recycling of some medical wastes prior to final disposal, if not infected or contaminated. Incineration could be used in medical waste treatment until another common treatment method and steam sterilization is available in near future. Therefore, toxic substances such as dioxin emissions at medical waste incinerators should be closely monitored to reduce potential risks to humans and the surrounding environment [29].

Lack of awareness, appropriate policy and laws, and apathy are responsible for improper management of medical waste in Dhaka City. The process of collection, segregation and disposal of medical waste is not performed according to recommended standards, and concerned people are exposed to the danger of such wastes. Safe disposal of medical waste is essential and is handled in a very professional way in many countries. The existing medical waste management system currently serves a limited number of HCE. New facilities should be established in different parts of the city or the existing facility should be expanded.

## List of abbreviations

BMCH: Bangladesh Medical College Hospital; BOD, Biochemical Oxygen Demand; CIDA, Canadian International Development Agency; COD, Chemical Oxygen Demand; DCC, Dhaka City Corporation; DMCH, Dhaka Medical College Hospital; DoE, Department of Environment; GoB, Government of Bangladesh; HCE, Health care Establishments; ICDDR, B International Centre for Diarrhoeal Disease and Research in Bangladesh; ICU, Intensive Care Units; MoEF, Ministry of Environment and Forest; OPD, Out-patient-departments; OT, Operation Theatre; PAH, Polycyclic Aromatic Hydrocarbons; PCDD/F, Polychlorinated Dibenzodioxin and Dibenzofuran; PPP, Public-Private Partnership; PRISM, Project in Agriculture, Rural Industry, Science and Medicine; RAJUK, RAJdhani Unnayan Kortipokkha (Capital Development Authority); TSS, Total Suspended Solids; WSP, Water and Sanitation Programme

## Competing interests

The author(s) declare that they have no competing interests.

## Authors' contributions

MMH was the principal investigator of this research. He made substantial contributions to conception, design, and drafting the manuscript with statistical analysis, mapping and interpretations. SAA made substantial contributions to conception, design, and drafting the manuscript with data interpretation. KAR participated in designing of the study & revising it critically for important intellectual content. TKB made contributions to design the manuscript with data collection and analysis. All authors read and approved the final manuscript.

## Pre-publication history

The pre-publication history for this paper can be accessed here:


